# Compliance of infant formula promotion on websites of Brazilian manufacturers and drugstores

**DOI:** 10.11606/s1518-8787.2020054001327

**Published:** 2020-02-05

**Authors:** Isabella Scatamacchia Cordeiro Ferraz Prado, Ana Elisa Madalena Rinaldi

**Affiliations:** I Universidade Federal de Uberlândia Faculdade de Medicina UberlândiaMG Brasil Universidade Federal de Uberlândia. Faculdade de Medicina. Curso de Nutrição. Uberlândia, MG, Brasil

**Keywords:** Infant Formula, legislation & jurisprudence, Guideline Adherence, Direct-to-Consumer Advertising, e-Commerce

## Abstract

**OBJECTIVE:**

To verify the compliance with Law No. 11,265/2006 in the promotion strategies for infant formula in Brazilian websites of manufacturers and drugstore networks.

**METHODS:**

This was a cross-sectional study conducted in 2017. We analyzed the compliance to attributes of the Law No.11,265/2006 (Law for Marketing of Foods for Infants and Toddlers, Feeding Bottles, Teats and Pacifiers) in five websites of infant formula manufacturers and nine websites of drugstore networks. The main attributes assessed were: the presence of drawings or representations of children, the presence of warning statements displayed in conspicuous and prominent spaces informing if products are intended for infants aged under or over 6 months, the adequate display of infant formulas/similar products, and the presence of pop-ups with other infant formulas or links to websites for children’s products. All compliances and non compliances verified were described in absolute and relative frequencies.

**RESULTS:**

We verified that 80% of the websites of infant formula manufacturers displayed advertisements for other children’s food products. The main non compliance in infant formula manufacturer’s websites was the absence of warning statements about products intended for infants over 6 months of age. Only 33% of the drugstores’ websites complied with Law No. 11,265/2006. The main non compliances in these websites were the absence of warning statements on products intended for infants over 6 months of age (100%), the presence of pop-up advertisements for other infant foods (77%) and the presence of advertisements for other children’s food products (92%).

**CONCLUSION:**

We identified non compliances with the Law No. 11,256/2006 in almost all websites of infant formula manufacturers and in all the websites of drugstore networks. Most promotion strategies were found at drugstore websites, which are the main channels for online sales.

## INTRODUCTION

In 1981, the World Health Assembly adopted the International Code of Marketing of Breast-milk Substitutes aiming to restrict inappropriate advertising and protect breastfeeding. It was the first actual attempt to fight the damaging effects of the marketing of human milk substitutes, feeding bottles and teats. Although the Code expresses the collective will of the membership of the world’s highest authority in health, with political and moral weight, after 34 years of its adoption, massive advertisement activities continue to undermine the efforts to increase breastfeeding rates^1^.

Along with the development of the Code, the International Baby Food Action Network (IBFAN) was created, with the main purpose of promoting and protecting breastfeeding, to benefit the health of infants and toddlers^2 , 3^. To achieve this goal, IBFAN conducts periodic monitoring to verify and report the compliance with the Code and to ensure more engagement of the industries, businesses and health professionals in self-regulation by the Code. IBFAN actively opposes any unethical advertisement or marketing action that may undermine breastfeeding^2^.

In Brazil, the Norm for Marketing of Breast-milk Substitutes (NBCAL), of 1988, was the first attempt to develop a document similar to the Code. After 13 years, parameters were introduced to regulate the production and marketing of feeding bottles, teats and pacifiers, and the National Health Surveillance Agency (ANVISA) changed the legal status of NBCAL, turning it into a regulation – Enforcement 2051 (2001)^4^, Resolutions 221^5^ and 222(2002)^6^. In 2006, NBCAL became Law No. 11,265^7^, and in 2015 it was officially regulated in Decree No. 8,552/2015^8^. In 2018, this Law was included in Decree No. 9,579, which describes the legal human rights of infants, children and adolescents^9^. It is noteworthy that, since 2006, the new legal status has allowed the application of sanctions for breaches, based on Law nº6437/1977^10^.

NBCAL prohibits any kind of advertisement for infant formula, follow-up formula, nutrient formula presented and/or indicated for high-risk newborns, feeding bottles, teats, pacifiers and nipple shields. For these products, commercial advertisements in any media is prohibited, including merchandising, written, audio or visual advertisements, and gifts, besides the most widely used media today, internet advertising^4^. The internet and the social media are currently used by manufacturers of breastmilk substitutes to promote their products. Studies on this topic are still incipient but likely to increase, as access to these media also increases^11 , 12^.

Despite the substantial apparatus in the Brazilian constitution and in international policies, the practice of breastfeeding has become fragile. This is aggravated by the huge amount of information on the internet on matters related to health, breastfeeding and complementary feeding. Currently, the internet is one of the largest and most important communication channels^12^.

The latest data on internet access in Brazil, from the National Household Sample Survey (PNAD) of 2016, showed that 69.3% of households (non-commercial) were connected to the internet, 97.2% of which through cell phones. The data also showed that in the previous three months, 64.7% of the 10-year-old Brazilians had used the internet, and 94.2% of them used it to send or receive written or audio messages or images^a^. The percentage of people aged between 15 and 74 years who made online purchases of goods and services was 16% in 2005 and 18% in 2008^b^.

The monitoring conducted by IBFAN Brazil is primarily focused on the products covered by NBCAL sold in physical shops. Due to the increased access to the internet and to the growth of online sales in Brazil, it is important to know whether there are sales or discounts on these products on the websites of commercial formula manufacturers and of companies that sell them online. Thus, our objective was to verify the compliance with Law No. 11,265/2006 in the promotion strategies on the websites of infant formula manufacturers and drugstore networks in Brazil.

## METHODS

### Study design

This is a cross-sectional study with the objective of analyzing the Brazilian websites of formula manufacturers and drugstore networks. This study was not submitted to Research Ethics Committees because all data are publicly accessible, complying with the National Counsil of Health, Resolution number 510 of 2016^c^.

From September to November 2017 we searched for the websites and the studied the current Legislation(Law No. 11,256/2006^4^ and Decree No. 8,552/2015^8^). We examined the promotion of formula for infants (up to six months), follow-up formula (infants aged six to 12 months) and formula for young children (child aged 12 months to 36 months old).

### Sample of study

Our sample was composed of websites of formula manufacturers and drugstore networks. The selection of major drugstore networks was based on the survey of the largest companies in the country, carried out by the Brazilian Retail and Consumption Society (SBVC) in 2017^d^. This survey ranked the top 300 Brazilian retail companies which had grown more than the average growth presented by the retail industry in 2017. For the present study, we selected the 15 largest drugstore networks, of ten retail groups. These companies were selected because they have physical stores in 26 Brazilian states (96.3%) and nationwide scope through thesales in their websites.

After this selection, we verified if the websites of each drugstore were designed for online sales. Subsequently, for the networks that had online shops, we entered the term “infant formula” in the search box, and examined the predefined elements appearing. We searched for products of the same manufacturers on all drugstore websites. The drugstores selected from the 2017 Ranking were: DrogaRaia^®^, Drogasil^®^, Farmasil^®^, Univers^®^, 4BIO^®^, Drogaria São Paulo^®^, Drogaria Pacheco^®^, Pague Menos^®^, Angeloni^®^, Farmalider^®^, Coop^®^, Drogaria São João^®^, Drogaria Araújo^®^, Panvel^®^ and Catarinense^®^. Out of these 15 networks, we chose the nine that had online shops. The Pague Menos^®^ website the required selection of a Brazilian State to access the website. In this case, we selected the State of Minas Gerais, where the study was conducted. In addition, the Catarinense^®^ network has a website for online sales called Farmagora^®^, in which the search was conducted.

We chose five infant formula manufacturers — Abbott Laboratories^®^, Biolab^®^, Danone^®^, Mead Johnson^®^, Nestlé^®^ — because they were available in all the websites of drugstore networks selected for our study. Four multinational companies (Abbott^®^, Danone^®^, Mead Johnson^®^ and Nestlé^®^) dominate the infant formula market globally^13^. Euromonitor provides data on the infant formula market and confirmed their dominance for the year of 2013^14^. However, since Euromonitor data are restricted, being necessary to pay to get access to complete data, we opted to use results from previous recent studies.

### Analyzed attributes of Law 11,265/2006

All data were extracted from the websites by the first author of this study and the data collection was conducted in three stages: 1) listing of all trademarks of infant formula sold in Brazil; 2) accessing the websites of each one, by their trade name, in the Portuguese language; 3) inspecting the conformity of marketing and promotion of infant formulas, according to IBFAN.

The quantitative and qualitative data collected were fully based in attributes of Law No. 11,265/2006^4^ and Decree No. 8,552/2015^8^, which are organized in the Form 5.2, “Analysis of promotional material,” of the Manual of IBFAN Monitoring Training Course^3^.

The attributes observed were: total number of formulas available on the websites; use of visual images of children (illustrations, pictograms, pictures/images of infants or babies) (yes/no); adequate promotion of infant formula or similar plant-based formula, (yes/no); presence of the required warning statement on the risks of food for babies under the age of six months (yes/no); presence of the required warning statement on the risks of food for babies over six months old (yes/no); warning statements displayed in a conspicuous and prominent space (yes/no); promotion of other infant foods or products (yes/no); pop-up advertisements that link to websites of other child supplies or formula retailers (yes/no); websites of infant formula manufacturers with interfaces for professionals different from those accessed by the public in general (yes/no); pop-ups with additional information on the products on the websites of drugstore networks (yes/no). The use of images of infants on formula packaging or websites is forbidden in Brazil, being seen as a strategy to persuade parents to buy the product.

We adopted the concept of “promotion” proposed by NBCAL, that is, “the set of informative and persuasive activities conducted by companies responsible for the production or manipulation, distribution and sale of a product, with the purpose of inducing the purchase or sale of it.” (author’s translation). The promotion of feeding formula for newborns and follow-up formula is forbidden. Formula for young children can be promoted if the following warning is written in bold capital letters: **“BREASTFEEDING PREVENTS INFECTIONS AND ALLERGIES AND IT IS RECOMMENDED UP TO TWO YEARS OF AGE OR BEYOND.”** Thus, it has become important to monitor websites of infant formula manufacturers, as well as those of drugstore networks, as they are the key distributors of these products.

According to the Cambridge Dictionary^e^, the US term “pop-up” means “a new window that opens quickly on a computer screen in front of what you are working on”, referring to any window that links to other websites.

### Data analysis

All data were entered into Excel^®^ spreadsheets. For each item, the word “no” was written when this aspect was not available, “yes” when it was present, and “Not Applicable – NA” when it did not apply to a particular item. The data were then summarized in absolute frequencies, indicating the total number of infant formulas available on the websites of manufacturers and drugstores, and in relative frequencies, summarizing the attributes that were inadequate/non compliant in the websites.

## RESULTS

For this study, we selected the websites of five infant formula manufacturers and nine drugstores. The websites of two out of five manufacturers , Nestlé^®^ and Danone^®^, had sections that described all the infant formulas available ( Table 1 ). When both websites were accessed, pop-ups appeared on the screen with a warning statement – as defined by the Brazilian legislation on the commercialization of these foods – with two options: “I want to proceed” and “I do not want to proceed.” By clicking the option “I want to proceed,” we could see the infant formulas sold in the country; 16 products in total. The mandatory warning notice for this kind of product could be seen in the product illustrations in small boxes on their labels and at the end of the website, but they were not highlighted in a prominent manner, as required by law.

**Table 1:** Description of attributes assessed in the websites of infant formula manufacturers. Brazil, 2017.

Attributes	Infant formula manufacturers				

	Abbott^®^	Biolab^®^	Danone^®^	Mead Johnson^®^	Nestlé^®^
Drawings/Representations of children	NA	NA	NO	NA	NO
Adequate offer of infant formulas or similar products	NA	NA	NA	NA	NA
Warning statements up to 6 months old	NA	NA	YES	NA	YES
Conspicuous warning statements	NA	NA	NO	NA	NO
Promotes other foods	YES	NO	YES	YES	YES
Warning statements for 6 months or older	NO	NA	YES	NO	YES
Conspicuously displayed warning statements	NA	NA	NO	NA	NO
Pop-ups	NO	NO	NO	NO	NO
Exclusive interface for professionals	NA	NA	YES	YES	YES
**Total of non compliances**	1	0	2	1	2

The other three manufacturer brands did not have a specific section for infant formulas on their websites. Abbott^®^ and Biolab^®^ websites did not mention this product line, even though the companies sell it in the country. The website of Mead Johnson^®^ provided a list of all infant formulas, but a notice explained that the company does not promote these products, complying with the legislation. In addition, when we selected “infant formula” products, we were taken to a website with access restricted to healthcare professionals. No manufacturer’s website allowed online purchase of their products. Danone^®^, however, was the only company that had links to shopping websites, 75% of which belonged to drugstore networks. On the websites that sold infant formulas, the area of access to these products, unlike the other accessible areas, had no images of children, as required by law. In addition, none of the websites had advertisements of the infant formulas or images of children ( Table 1 ). Of the five websites analyzed, four promoted other children’s foods, but not as specified by legislation ( Table 1 ).

The compulsory warning statement for most of these products was not clearly displayed. On the Nestlé^®^ website, the icon “access the website” overlapped the warning box. The warning statement on the website of Danone^®^ was at the end of the page, far from the product illustrations. Mead Johnson^®^ displayed the statement along with the preparation recommendations, in the promotional material of one of its products, in a case smaller than required and physically distant from the information. The Biolab^®^ website was the only one that did not display information about their infant formulas. The total of non compliances is shown in Table 1 .

The kind and number of non compliances of drugstore websites are described in Table 2 . All drugstore websites showed some kind of non compliance. In the drugstore networks selected for our study, there was a great variety of products under the same brand of infant formula. Of all drugstores examined, we found that 66.6% did not conform to the requirement of displaying a mandatory warning statement on the feeding of babies aged up to six months and 100% did not display warnings on the feeding of babies aged six months and older ( Table 2 ).

**Table 2:** Attributes observed in the websites of drugstore networks. Brazil, 2017.

Attributes found	Droga Raia^®^	Drogaria Araújo^®^	Drogaria Catarinense^®^	Drogaria São Paulo^®^	Drogaria Pacheco^®^	Drogasil^®^	Farmácia Angeloni^®^	Farmácias Pague Menos^®^	Panvel Farmácias^®^
Drawings/ Representations of children	NO	NO	NO	NO	NO	NO	NO	NO	NO
Adequate offer of infant formulas or similar products	YES	NO	NO	YES	NO	YES	NO	NO	NO
Warning statements up to 6 months old	YES	YES	NO	NO	NO	YES	NO	NO	NO
Conspicuous warning statements	YES	YES	NA	NA	NA	YES	NA	NA	NA
Promotes other foods	YES	YES	YES	YES	YES	YES	YES	YES	YES
Warning statements for 6 months or older	YES	YES	NO	NO	NO	YES	NO	NO	YES
Conspicuously displayed warning statements	YES	YES	NA	NA	NA	YES	NA	NA	YES
Pop-ups with additional product information	NO	NO	NO	NO	NO	NO	NO	NO	NO
Pop-ups of other products for children	YES	YES	NO	YES	YES	YES	YES	NO	YES
**Total of non compliances**	2	2	2	3	5	2	5	4	3

**Figure 1:**
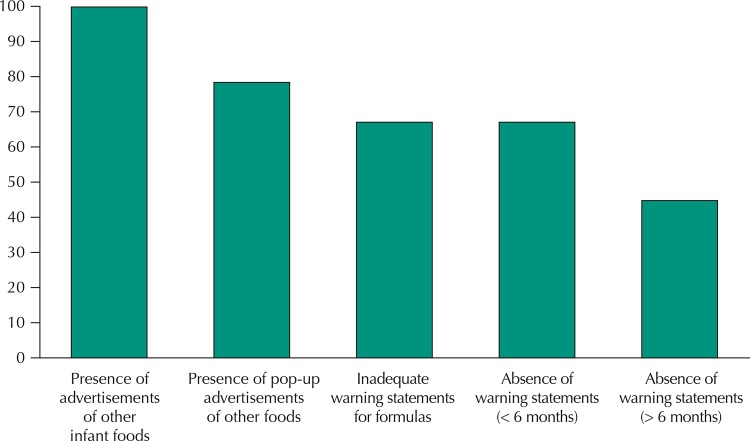
Non conformity (%) of infant formulas on the drugstores’ websites. Brazil, 2017.

During our search, the Pacheco^®^ website announced a price reduction for one of the infant formulas available, disregarding current legislation. The warning statement was displayed, but not in a conspicuous manner, as stipulated by the law. Another important issue observed during data collection was the absence of images of children on all drugstore websites ( Table 2 ). However, we observed that for other foods, such as milk-based foods, complementary foods, and infant purees and products, there was an excessive use of images of children and wording designed to persuade parents to buy these food products for their children. Regarding the promotion of other foods on the drugstore websites, the mandatory warning statement only appeared in 44.4% of them ( Table 2 ). We observed different treatments of infant formula in manufacturers’ and drugstores’ websites. For example, Biolab^®^ does not display these products on its own website, but on drugstore websites we found advertisements for its new rice-based infant formula which did not show the warning statement as specified. Figure 1 summarizes the non compliances found in the drugstore websites.

When conducting the research, we found a considerable difference between the number of infant formulas shown in the websites of drugstores and manufacturers ( Table 3 ). On the Danone^®^ website, there were 16 different types of infant formulas, while on the drugstores’ websites there were 19 products of this brand. In addition, the Nestle^®^ website showed 16 kinds of infant formula available, whereas on the drugstores’ websites we found 24 products of this same brand. The websites of manufacturers Biolab^®^, Abbot^®^ and Mead Johnson^®^ did not have lists of the available formulas. However, on the drugstore websites two types of infant formula produced by Biolab^®^, six by Abbot^®^ and nine by Mead Johnson^®^ were identified. Thus, the number of products listed is higher in the drugstore networks’ websites.

**Table 3:** Total number of infant formulas per website. Brazil, 2017.

Websites	Total
*Manufacturers*	
*Abbott* ^®^	0
*Biolab* ^®^	0
*Danone* ^®^	16
*Mead Johnson* ^®^	0
*Nestlé* ^®^	16
*Drugstores*	
*Droga Raia* ^®^	57
*Drogaria Araújo* ^®^	48
*Drogaria Catarinense* ^®^	57
*Drogaria São Paulo* ^®^	80
*Drogaria Pacheco* ^®^	79
*Drogasil* ^®^	57
*Farmácia Angeloni* ^®^	13
*Farmácias Pague Menos* ^®^	38
*Panvel Farmácias* ^®^	62

## DISCUSSION

In this study, we found at least one non compliance with the legislation on the websites of infant formula manufacturers. None of these websites promoted these products, and two of them used a pop-up notice to inform consumers that they do not promote infant formulas. They also did not sell products online, but provided links to other websites that sold formulas. Most non compliances were identified at drugstore websites, maybe because the online sales are their main purpose. We found a larger variety of infant formulas on these websites compared to the websites of manufacturers, 100% of them promoted other children’s foods and only four out of nine drugstores (44.4%) included the mandatory warning statements for these products.

The Law No. 11,265/2006 forbids the promotion of food products for infants, but allows the promotion of products for young children if there is a statement in them about breastfeeding. We believe that this recommendation does not support the parents and the society to continue breastfeeding practices after 12 months.

The analysis presented in this study is still not found in the literature, but it is well established that there are new means of promotion using the Internet. A report by the WHO mentions that in Brazil infant and young children foods are promoted on social media because the current restrictions do not cover this means of communication^1^. As aforementioned, Law No. 11,265/2006^4^ and Decree No. 8,552/2015^8^ provide the applications of sanctions for breaches. However, they have no specific description of online sales on websites of drugstores, supermarkets, formula manufacturers or other establishments that display these products. Decree No. 9,579/2018^9^, prohibits the commercial promotion of formulas for high risk newborns, infant formulas, follow-up formulas, and powdered milks, in any means of communication, including indirect or hidden advertisements, and announcements by electronic, written, audio and visual means. In our interpretation, the irregularities found in this study fit into indirect advertising both by infant formula manufacturers and drugstores. Manufacturers, distributors and importers are responsible for informing its commercial representatives and hired advertising agencies about the conditions of this Decree^8^.

In the US, advertisement of infant formulas was identified on social media such as *Facebook* , *Twitter* , cell apps, *My Space, Google+* , and *YouTube* , as well as in sponsored comments on blogs and websites of trademarks. Ten out of eleven of these trademarks had a presence on social media. There was also social media presence on the manufacturers’ websites. The target audience of the messages being promoted was composed of pregnant women and mothers. It was possible to interact with other people, sharing information and leaving reviews of products, in addition to other comments and users’ advises on infant feeding^15^. This kind of marketing somehow exempts companies from the compliance with the legislation, since it is not the company itself that advertises, but the established social media.

Actions should be taken against any non compliance that is considered “capable of revolutionizing the entire field of communication as well as the economy” as established by Law No. 11,265/2006. In this case, the manufacturers and distributors are responsible for failing to meet the legal requirements for the promotion of their products. It is likely that the exposure, sales, and price reductions of infant formulas in drugstores are defined by manufacturers. Promoting and supporting breastfeeding and child health are the goals of IBFAN, so it should contact contravening companies and inform them of the need to end unethical marketing strategies.

Abrahams (2012)^15^ emphasized the need for institutions engaged in promoting and protecting breastfeeding to pay special attention to new promotion strategies developed by manufacturers of human milk substitutes, monitoring them on social media.

In 2014 alone, the brands Nestlé^®^, Danone^®^, Mead Johnson^®^ and Abbot^®^ together controlled 55% of the global market of infant formula. It is a profitable product, and if we assume a 10–20% net profit, we can estimate that by 2019 the global market for infant formula would have reached $US 70.6 billion with an expenditure of $US 4–6 billion per year in the promotion of these products. On the other hand, the world gross economy loses about $US 302 billion annually due to nutritional deficiency^11 , 16 , 17^.

Independently of the media used in the promotion, it can involve strategies described in the literature, like cross-promotion. This marketing practice makes it possible to use promotional means and activities in the specific environments of one product to advertise another without the costumer noticing^1^. This practice occurs due the loopholes in the current legislation. In order to decrease the promotion of these products, other items that are not currently covered could be included in the regulations^18^.

We identified three main limitations of our study. The first is that we restrained our website search to the biggest drugstores, using the 2017 SBVC Ranking. There are other smaller drugstores that sell products on their websites which we did not analyze. The second is the impossibility of assessing the percentage of products sold through the websites of manufacturers and drugstore networks. The last is the impossibility of assessing the percentage of pregnant women and mothers that access these two sources of formula: the websites of infant manufacturers and drugstores.

It is possible to see the promotion of infant formula both on the websites of manufacturers and on those of retail drugstore networks. Regarding the manufacturers, although there were warning statements in pop-ups when we searched for infant formulas in them, attention was drawn to the products. Besides that, even if there were no images of children associated to the infant formulas shown on manufacturers’ or drugstores’ websites, the attraction related to these images in other foods could interfere with the mothers’ decision to breastfeed.

## CONCLUSIONS

Our study showed that almost all the websites of infant formula manufacturers presented some kind of non compliance with the Law No. 11,265/2006, and all websites of drugstores showed at least two non compliances. The main non compliance identified on these websites was connected to advertising and sales. Our results are relevant, helping policy markers to monitor and evaluate the compliance with Law No. 11,265/2006, which protects the mothers’ right to breastfeed, and preserves all the population from commercial advertisement which can distort their decisions about the use of infant formula.

## Footnotes

a Instituto Brasileiro de Geografia e Estatística. Pesquisa Nacional por Amostra de Domicílio – PNAD Contínua. Acesso à Internet e à televisão e posse de telefone móvel celular para uso pessoal 2016. Rio de Janeiro: IBGE; 2016 [cited 2018 Dec 18]]. Available from: https://loja.ibge.gov.br/pnad-continua-acesso-a-internet-e-a-televis-o-e-posse-de-telefone-movel-celular-para-uso-pessoal-2016.html

b United Nations, Economic Commission for Latin America and the Caribbean. CEPALSTAT: bases de dados y publicaciones estadísticas. Santiago de Chile; cited 2017 Dec 26. Available from: http://interwp.cepal.org/sisgen/SisGen_MuestraFicha.asp?indicador=1883&id_estudio=280

c Conselho Nacional de Saúde. Resolução Nº 510, de 7 de abril de 2016. Dispõe sobre as normas aplicáveis a pesquisas em Ciências Humanas e Sociais cujos procedimentos metodológicos envolvam a utilização de dados diretamente obtidos com os participantes ou de informações identificáveis ou que possam acarretar riscos maiores do que os existentes na vida cotidiana, na forma definida nesta Resolução. Diario Oficial Uniao. 24 maio 2016; Seção 1:44.

d Sociedade Brasileira de Varejo e Consumo. As 300 maiores empresas do varejo brasileiro. São Paulo, SP: SBVC; 2017 [cited 2017 Aug 10]. Available from: http://sbvc.com.br/ranking-sbvc-as-300-maiores-empresas-do-varejo-brasileiro-2017/

e Cambridge Dictionary. Meaning of “Pop-up.” Cambrige (UK): Cambridge University Press; cited 2019 Dec 20. Available from: https://dictionary.cambridge.org/pt/dicionario/ingles/pop-up
